# Drug Release from a Spherical Matrix: Theoretical Analysis for a Finite Dissolution Rate Affected by Geometric Shape of Dispersed Drugs

**DOI:** 10.3390/pharmaceutics12060582

**Published:** 2020-06-23

**Authors:** Yung-Sheng Lin, Ruey-Yug Tsay

**Affiliations:** 1 Department of Chemical Engineering, National United University, Miaoli 36063, Taiwan; 2 Department of Biomedical Engineering, National Yang-Ming University, Taipei 11221, Taiwan; 3 Center for Advanced Pharmaceutics and Drug Delivery Research, National Yang-Ming University, Taipei 11221, Taiwan

**Keywords:** drug release, dissolution, diffusion, Higuchi’s model, shape

## Abstract

Amending the neglect of finite dissolution in traditional release models, this study proposed a more generalized drug release model considering the simultaneous dissolution and diffusion procedure from a drug-loaded spherical matrix. How the shape factor (*n* = 0, 1/2, and 2/3 for the planar, cylindrical, and spherical geometry, respectively) of dispersed drug particles affected the release from the matrix was examined for the first time. Numerical solutions of this generalized model were validated by consensus with a short-time analytical solution for planar drugs and by the approach of the diffusion-controlled limits with Higuchi’s model. The drug release rate increases with the ratio of dissolution/diffusion rate (*G*) and the ratio of solubility/drug loading (*K*) but decreases with the shape factor of drug particles. A zero-order release profile is identified for planar drugs before starting the surface depletion layer, and also found for cylindrical and spherical dispersed drugs when *K* and *G* are small, i.e. the loaded drug is mainly un-dissolved and the drug release rate is dissolution-controlled. It is also shown that for the case of a small *G* value, the variation of drug release profile, due to the drug particle geometry, becomes prominent. Detailed comparison with the results of the traditional Higuchi’s model indicates that Higuchi’s model can be applied only when *G* is large because of the assumption of an instantaneous dissolution. For *K* = 1/101–1/2, the present analysis suggests an error of 33–85% for drug release predicted by Higuchi’s model for *G* = 10^0^, 14–44% error for *G* = 10^1^, while a less than 5% error for *G* ≧ 10^3^.

## 1. Introduction

A reservoir-type drug carrier with un-dissolved drug particles loaded uniformly in a matrix is one of the most common formulations for a controlled release system. However, various mathematical models have been proposed to describe the drug release from this system over the years. Higuchi’s model proposed in the early 1960s remain the best known and most popular one [1,2,3]. In his model, a moving front separates the inner region coexisting dissolved and un-dissolved drugs from the outer region of depletion layer, where the un-dissolved drug is exhausted and the concentration of dissolved drug is analyzed by a pseudo-steady state approximation [4,5]. The assumption of instantaneous dissolution in Higuchi’s model simplifies the release system to become a purely diffusion-controlled release of dispersed drugs. 

After Higuchi’s model, many works directed towards various improvements to extend its applications [6,7,8]. In spite of these further refinements, the effect of a finite dissolution rate is still ignored in general. Nevertheless, quite often it is found that drug release is also controlled by the dissolution process of the dispersed drug particles in the matrices [9,10]. The effects of a finite dissolution rate have been observed for rather insoluble substances [11,12]. It is, therefore, necessary to develop a more general dissolution-diffusion model to describe release behavior.

In the late 1970s, the issue of a finite dissolution rate on drug release was first examined for simple suspended drugs [13,14]. Later on, the problem was extended to systems loaded with un-dissolved drugs in a supporting matrix in the 1980s [15,16,17]. Drug release problems of this kind can be described by incorporating a drug dissolution term into the Fickian diffusion equation. However, due to the complexity of the dissolution/diffusion-controlled drug release system, only solutions for special cases, such as for drug release from a semi-infinite matrix [15], a thin film [16], or at the early stage before the formation of a surface depletion layer [17], were examined. There are few reports associated with this scope until the early 2000s [18]. After that, Frenning published three papers in series to analyze the release of slowly dissolving drugs from a planar matrix [19], a spherical matrix [20], and a cylindrical matrix [21]. The system was formulated by two coupled nonlinear partial differential equations describing the drug dissolution and diffusion processes and was solved numerically for the dynamic drug release curves. Cabrera and Grau [22,23] used Green’s functions to solve the dissolution/diffusion-controlled drug release by differential mass balance equations governing the simultaneous dissolution and diffusion process. However, both these two groups assumed solid drug particles were invariably spherical in shape.

In the mathematical formulation for drug release, drug dissolution acts as a source term in the Fickian diffusion equation and is universally described by the Noyes–Whitney equation [24]. However, the formulation of this dissolution term is still indefinite because of the unclear surface area of solid drugs. Currently, there are two perspectives proposed for the available surface area of dispersed drugs during dissolution. One is that the surface area is assumed to be proportional to the drug volume and described as the power 2/3 of the solid drug concentration, i.e., the drug particle is assumed to be spherical and retains its shape during the process of drug dissolution [19,20,21,22,23,25,26]. The other assumes the surface area remains constant during the dissolution process [13,14,15,16,17]. Obviously the relationship between the surface area and solid drug concentration depends on the geometry of the un-dissolved drug particles, and it will affect the drug release profiles. 

The error of drug release rate estimated by the classic Higuchi’s model for a dissolution/diffusion coupled system has never been closely examined. To develop a more accurate and generalized model, thus, continuously gains attention [18,27,28,29]. This study constructed a more generalized dissolution-diffusion model for drug release from a spherical matrix system loaded with dispersed un-dissolved drug particles of different shapes. The effect of the geometrical shape of the dispersed drug particles on the drug release profile was examined for systems with different *G* (the ratio of dissolution rate/diffusion rate) and *K* (the ratio of solubility/total drug loading value), respectively. Furthermore, to justify the general applications of Higuchi’s model in a reservoir type, errors introduced by the assumption of instantaneous dissolution in Higuchi’s model were analyzed in detail. 

## 2. Methods

### 2.1. Mathematical Formulation

For the system of a reservoir type drug-containing non-swellable spherical carrier, dissolved drug molecules and un-dissolved dispersed drug particles are spread uniformly in the matrix. The presumed mechanism of drug release is that the dispersed drug must dissolve first in the matrix and then diffuse outward to a perfect sink medium. Because of the spherical symmetry, concentrations of the dispersed drug *C_a_* and dissolved drug *C* are functions of the radial coordinate *r* and time *t* only. The drug dissolution rate *S(r, t)* is described by the Noyes–Whitney equation [24] and formulated as a function of the surface area *A* of dispersed drugs and the concentration difference between the drug solubility *C_s_* and *C*. Therefore,

*S*(*r,t*) = *kA*(*C_s_ − C*) (1)

where *k* is the dissolution rate constant with the unit of area^−1^ time^−1^. By the proportion expression, the surface area can be described by the power law of *C_a_* and expressed as

*A* = *A_o_*(*C_a_/C_ao_*)*^n^* (2)

where *A_o_*, *C_ao_,* and *n* are the initial drug surface area per unit volume of sphere, the initial dispersed drug concentration, and the geometry-dependent factor of dispersed drugs, respectively. Assume only the thickness or radius of the drug particles decreases during the release process. It can be derived that the shape factors for planar, cylindrical, and spherical drugs are 0, 1/2, and 2/3, respectively. Applying Fick’s second law of diffusion and mass balances for dissolved and dispersed drugs, one obtains the following coupled governing equations of the dissolution-diffusion release model for a spherical matrix:

(3a) $$\frac{\partial C}{\partial t}=D\left[\frac{\partial^{2}C}{\partial r^{2}}+\frac{2}{r}\frac{\partial C}{\partial r}\right]+kA_{0}{\left(\frac{C_{a}}{C_{a0}}\right)}^{n}(C_{s}-C)\left[1-u(r-r^{*})\right]$$

(3b) $$C_{a}=C_{ao}-\int_{0}^{t}kA_{0}{\left(\frac{C_{a}}{C_{a0}}\right)}^{n}(C_{s}-C)\left[1-u(r-r^{*})\right]dt$$

where *D* is the drug diffusion coefficient and *u*(r − r^*^) is a unit step function. r^*^ in the unit step function indicates the location of the moving boundary of the depletion zone, where the un-dissolved drugs are exhausted. 

Before solving the problem, the following non-dimensional variables are introduced:

*φ* = *C/C_t_* and *ϕ* = *C_a_/C_t_* (4)

*τ* = *Dt/r_o_^2^* and *η* = *r/r_o_* (5)

where *r_o_* is the radius of the spherical matrix and *C_t_*, defined as the sum of *C_s_* and *C_ao_*, is the initial drug loading. By substituting these dimensionless parameters into the coupled governing equations for the drug diffusion and dissolution processes, Equation (3) can be transformed into

(6a) $$\frac{\partial \varphi }{\partial \tau }=\frac{1}{\eta }\frac{\partial^{2}}{\partial \eta^{2}}(\eta \varphi )+G{\left(\frac{\phi }{1-K}\right)}^{n}(K-\varphi )\left[1-u(\eta -\eta^{*})\right]$$

(6b) $$\phi =(1-K)-\int_{0}^{\tau }G{\left(\frac{\phi }{1-K}\right)}^{n}(K-\varphi )\left[1-u(\eta -\eta^{*})\right]d\tau$$

where h^*^ = r^*^/r_o_, *K = C_s_*/*C_t_*, and *G = kA_o_r_o_^2^/D*. *K* is the ratio of solubility/drug loading and *G* is a dimensionless number representing the ratio of dissolution rate/diffusion rate. For a diffusion-controlled system, the dissolution rate is much larger than the diffusion rate and therefore G >> 1. It is assumed that saturated dissolved drugs appear originally, and, therefore, initial conditions can be expressed as

(7a) φ(0,η)=K

(7b) ϕ(0,η)=1−K

Applying symmetric and the perfect sink conditions at the center and surface of the drug carrier, respectively, one obtains the following boundary conditions:

(8a) $${\frac{\partial \varphi }{\partial \eta }|}_{\eta =0}=0$$

(8b) φ(τ,1)=0

### 2.2. Critical Time for the Formation of a Surface Depletion Zone

Let *τ_c_* be the critical time for the formation of a surface depletion zone. It is the time at which un-dissolved drugs at the sphere’s surface are exhausted. Before the formation of a surface depletion zone, by introducing the boundary condition of *φ* (*τ*,1) = 0 in Equation (8b) and the initial condition of *ϕ* (0,*η*) = 1 − *K* in (7b) into the governing equation of Equation (6b), the concentration of un-dissolved drugs at sphere’s surface, *ϕ* (*τ*,1), can be obtained, i.e.,

(9) $$\phi (\tau ,1)={\left[{\left(1-K\right)}^{1-n}-\frac{G}{{\left(1-K\right)}^{n}}(1-n)K\tau \right]}^{1/(1-n)}$$

For *τ* = *τ_c_*, *ϕ*(*τ*,1) = 0, one thus obtains

(10) $$\tau_{c}=\frac{\left(1-K\right)}{GK(1-n)}$$

**Figure 1:**
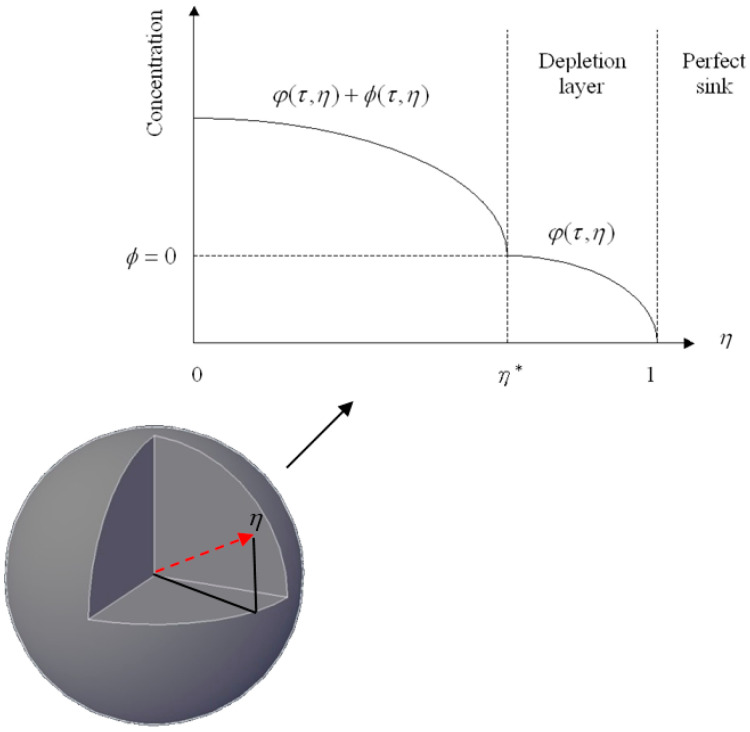
A schematic diagram of the drug concentration profile in a sphere immersed in an infinite medium.

Therefore, *τ*_c_ = (1 − *K)/GK*, 2(1 − *K)/GK* and 3(1 − *K)/GK* for planar, cylindrical, and spherical drug particles, respectively. For *τ* < *τ_c_*, the drug release process remains a fixed boundary problem. However, for *τ* > *τ_c_*, the boundary of the depletion zone proceeds inward with time and the drug release process turns into a moving boundary problem. Figure 1 shows a schematic diagram of the concentration profile described in the model for *τ* > *τ_c_*. Here, the moving boundary h^*^, defined by the dimensionless radius of the un-depleted zone, separates a depletion zone containing only dissolved drugs from a region containing both dissolved and un-dissolved drugs. 

### 2.3. Special Case of Planar Drugs

For planar drug particles, the shape factor *n* = 0 and prior to the formation of the depletion layer, h^*^ = 1 and the unit step function *u(η* − η^*^) in Equation (6) equals zero. Therefore, the drug release problem described in Equation (6) becomes linear, i.e.,

(11a) $$\frac{\partial \varphi }{\partial \tau }=\frac{1}{\eta }\frac{\partial^{2}}{\partial \eta^{2}}(\eta \varphi )+G(\frac{\phi }{1-K})(K-\varphi )$$

(11b) $$\phi =(1-K)-\int_{0}^{\tau }G(\frac{\phi }{1-K})(K-\varphi )d\tau$$

The analytic solution of this special case of the boundary value problem was first solved by Harland et al. [17], which gives the expressions of the final solutions of *φ*(*τ*, *η*) and *ϕ*(*τ*, *η*) as

(12a) $$\varphi (\tau ,\eta )=2\sum_{m=1}^{\infty }\frac{{\left(-1\right)}^{m+1}}{m\pi \eta }\sin(m\pi \eta )\left[\frac{G}{G+{\left(m\pi \right)}^{2}}+\frac{{\left(m\pi \right)}^{2}}{G+{\left(m\pi \right)}^{2}}e^{-[1+{\left(m\pi \right)}^{2}/G]\tau }\right]$$

(12b) $$\phi \left(\tau ,\eta \right)=\left(1-K\right)-\left[\tau +2\sum_{m=1}^{\infty }\frac{{\left(-1\right)}^{m}}{m\pi \eta }\sin\left(m\pi \eta \right)\left(\frac{G\tau }{G+{\left(m\pi \right)}^{2}}-\frac{{\left(m\pi \right)}^{2}}{G{\left(1+{\left(m\pi \right)}^{2}/G\right)}^{2}}(e^{-[1+{\left(m\pi \right)}^{2}/G]\tau }-1)\right)\right]$$

### 2.4. Numerical Solutions 

All calculations were performed using MATLAB programs (MathWorks Inc., USA). The PDEPE routine was applied to obtain the numerical solution of Equations (6). In this routine, a spatial discretization technique developed by Skeel and Berzins [30] was applied to transform the partial differential equation into an ordinary differential equation. Time integration using routine ODE15S was then employed to solve the resulting ordinary differential equation. The number of mesh points required for convergence of the numerical solution depended upon the *G* value. Convergence tests indicated that the larger the *G* value, the greater the need for more spatial mesh points. To have an acceptable accuracy, a fixed number of spatial mesh points, 2000, was applied for all *G* values. The relative error tolerance was set to 1 × 10^−3^ in PDEPE. The fraction of released drugs was obtained by evaluating the integral of the concentration profile, 1 − *φ* − *ϕ*, using the QUAD routine which controlled the relative error tolerance fixed at 1 × 10^−6^. The accuracy of the numerical evaluation of the infinite sums in Equation (12) was controlled at 1 × 10^−4^ for all time.

### 2.5. Higuchi’s Model

On the basis of an instantaneous dissolution assumption and the presence of a pseudo-steady state drug release at the surface depletion zone, Higuchi’s equation gave the following expression for drug release from a spherical matrix immersed in a perfect sink [5]:

(13) $$1+2{\left(\eta^{*}\right)}^{3}-3{\left(\eta^{*}\right)}^{2}+K\left[4{\left(\eta^{*}\right)}^{2}+\ln\frac{1}{\eta^{*}}-1-\eta^{*}-2{\left(\eta^{*}\right)}^{3}\right]=6K\;\tau$$

Here, the concentration of drugs beyond the surface depletion zone was assumed to be constant, and the drugs remaining in the depleted zone could be obtained by integrating the dissolved drug concentration from η^*^ to 1. Therefore,

(14) $$\mathrm{Fraction}\;\mathrm{of}\;\mathrm{released}\;\mathrm{drugs}=1-{\left(\eta^{*}\right)}^{3}-\int_{\eta^{*}}^{1}\varphi \cdot 3\eta^{2}\cdot d\eta$$

## 3. Results and Discussion

### 3.1. Validation of the Model

**Figure 2:**
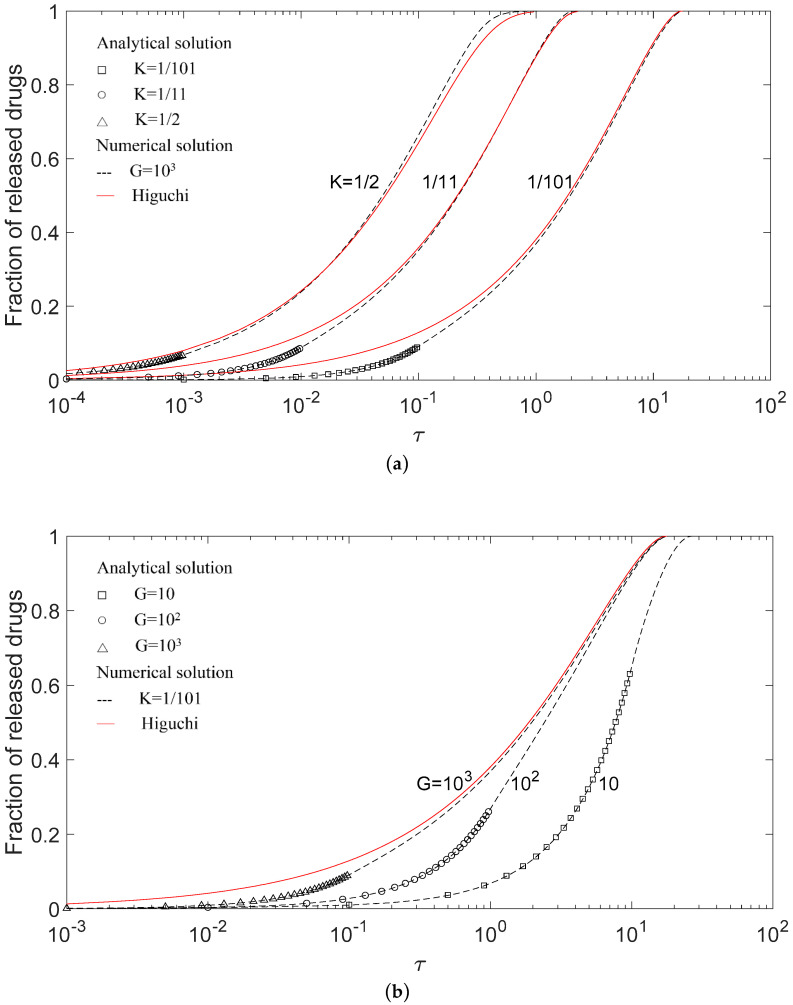
Comparison between Higuchi’s model and this present model for (**a**) *G* = 10^3^ with different *K*, from right to left, *K* = 1/101, 1/11, and 1/2, and (**b**) *K* = 1/101 with different *G* values from right to left, *G* = 10, 10^2^, and 10^3^. The marker is the analytical solution.

The drug release model in this study was verified for the special case of *n* = 0. Drug release profiles of numerical and analytical solutions in this work were compared with Higuchi’s model for (a) a fixed *G* with a different *K*, and (b) a fixed *K* with a different *G* in Figure 2. The markers represent analytical solutions describing the release before the formation of a depletion zone, and the solid lines and dash lines, respectively, represent the solution by Higuchi’s model and numerical solutions obtained herein. Because the closed form analytical solution can only be obtained before the formation of the depletion zone, it is noticed that analytic solutions are shown only before *τ_c_*, i.e., 10^−1^, 10^−2^, and 10^−3^ for *K* = 1/101, 1/11, and 1/2, respectively, in Figure 2a; and 10, 1, and 10^−1^ for *G* = 10, 10^2^, and 10^3^, respectively, in Figure 2b. The exact correspondence of markers to the lines indicates excellent agreement between the analytical and numerical solutions and suggests the accuracy of the numerical solutions. Besides, it is instructive to show the analytical solutions covering the major part of the release process for a small *G* value because a small *G* value represents relatively slow dissolution, and the depletion layer will occur in the late release process. The results also show that the effect of *K* values on applicable ranges of analytical solutions in the release process were slight.

The release profiles of different *G* values in Figure 2b differed from each other. With increasing *G* values, the drug release rate increased and the release profile approached that of Higuchi’s model. In other words, the drug release curves in Figure 2b will be insensitive to the dissolution effect when the *G* value is large. Higuchi’s solution stands for the limiting case of the *G* value approaching infinity and can be regarded as an asymptotic behavior of *G* values. Furthermore, numerical solutions in this work showed an initial delay compared with Higuchi’s model. The delay was due to the finite dissolution rate, and it was more conspicuous for slowly dissolving drugs in Figure 2b and enhanced by a high initial drug loading in Figure 2a.

Figure 2a shows that the deviation between the release profiles in Higuchi’s model and this work does not apparently decrease with the decrease in *K* values. The explanation for this is that the *G* value of 10^3^ used in Figure 2a is still not large enough for Higuchi’s model to work. On the other hand, it is somewhat surprising that Higuchi’s solution for a *K* value as large as 1/2 is not very different from the exact numerical solution as shown in Figure 2a. This suggests that Higuchi’s formula might also be applicable to drug release systems without a high drug loading ratio, and the accuracy of Higuchi’s model is more sensitive to the *G* value and less sensitive to the *K* value.

### 3.2. Effects of the Drug Shape Factor 

**Figure 3:**
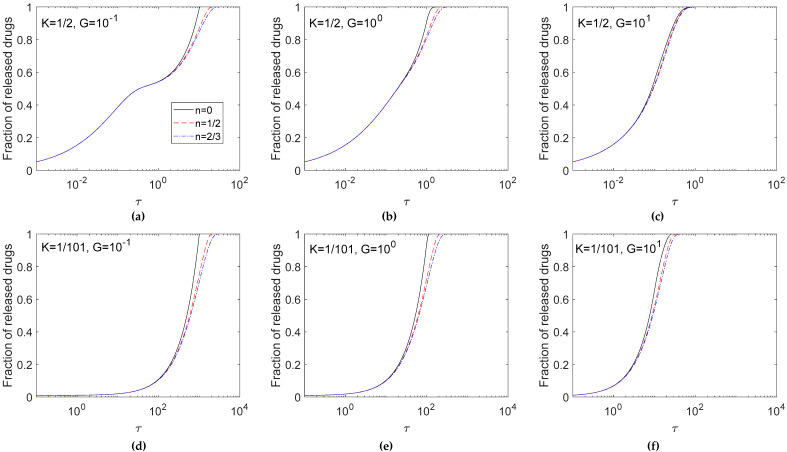
Effect of *n* on release fractions for *K* = 1/2 (**a**–**c**) and *K* = 1/101(**d**–**f**). Calculations are carried out for *G* = 10^−1^ (**a**,**d**), 10^0^ (**b**,**e**) and 10^1^ (**c**,**f**).

After verifying the exactitude of the numerical solutions, Figure 3 explores the effects of the shape of un-dissolved drug particles, i.e. *n* values, on the release profile. Calculations were performed for *n* = 2/3, 1/2, and 0, representing the spherical, cylinder, and planar shaped un-dissolved drug particles dispersed in the matrix, respectively for *G* = 10^−1^, 10^0^, and 10^1^ in the cases of *K* = 1/2 (Figure 3a–c) and *K* = 1/101 (Figure 3d–f). As a whole, the release profile displayed a sinusoidal shape of increase with the logarithm of time. The effects of *n* values became conspicuous in the late stage of the release profile.

Considering the surface area of solid drugs for dissolution in Equation (2), there is a fast decrease in the surface area with decreasing concentration of solid drugs in the release process for a large *n*. As shown in Figure 3, the case of *n* = 2/3 had the slowest release rate among the three *n* values due to the smallest surface area provided for dissolution during the release process. Therefore, the slower the dissolution rate was, the slower the release rate became. The effect of *n* values was conspicuous for a small *G* value, because for a large *G* value the drug release process was diffusion-controlled.

For systems with the same diffusivity, a larger *G* value indicates a higher dissolution rate and results in a faster exhausting rate as observed in Figure 3a–c for *K* = 1/2 and Figure 3d–f for *K* = 1/101. Among these three *G* values, *G* = 10^1^ (Figure 3c,f) indicated a quick increase in the fraction of released drugs, and the time required to exhaust the entirety of the loaded drugs was shorter than in the case where *G* = 10^−1^ (Figure 3a,d). Furthermore, the effects of *K* show that with the decrease in the *K* value, the high un-dissolved drug ratio decreased the drug release rate, and it took a longer time to exhaust drugs within the matrix. 

**Figure 4:**
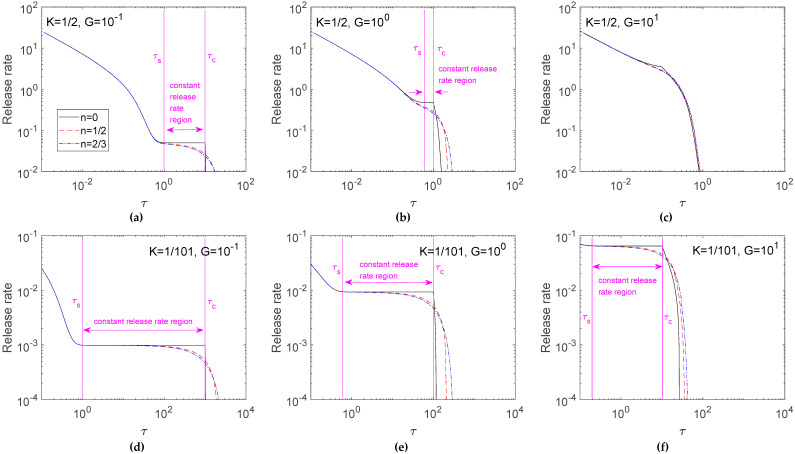
Effect of *n* on release rates for *K* = 1/2 (**a**–**c**) and *K* = 1/101(**d**–**f**). Calculations are carried out for *G* = 10^−1^ (**a**,**d**), 10^0^ (**b**,**e**) and 10^1^ (**c**,**f**).

Figure 4 further investigates the release rate in Figure 3. Results indicated that a more intense release rate was found for the early release profile, and the release rate decreased with time. For *K* = 1/2 (Figure 4a–c), half the loaded drugs were dissolved; therefore, the result of a fast release rate for a large *G* only occurred in late release time. For *K* = 1/101 (Figure 4d–f), the high ratio of un-dissolved drugs brought about an obvious dissolution effect that the release rate increased with the *G* value. On the other hand, the difference among three cases of *n* was small in early release time, but the deviation enlarged in late release time. This phenomenon was enhanced for a small *K*.

Results discussing the effects of the shape factor of the solid drugs were given by the release curve (Figure 3) and its corresponding release rate (Figure 4). These figures give data for *G* values of 10^−1^ to 10^1^ only. Results for *G* > 10^1^ were not shown because the shape factors of the solid drug particles no longer had a sensitive parameter for drug release in a diffusion-controlled system. From the release rate data, an interesting constant release rate region can be found for planar drug particles, and the effects of the shape factor on the release rate for *G* values of 10^−1^ to 10^5^ and *K* values of 1/2 to 1/101 were further analyzed quantitatively in the following section.

### 3.3. Identification of Constant Release Rate Region

The starting time for the moving front to proceed inward from the surface happened when η^*^ departed from the unity. As depicted in Equation (10), the *τ_c_* occurred at 1/*G*, 2/*G*, and 3/*G* in *K =* 1/2; and *τ_c_* = 100/*G*, 200/*G*, and 300/*G* in *K =* 1/101 for *n* = 0, 1/2, and 2/3, respectively. The *τ_c_* occurred obviously at the turning point in the release rate profile for the case of *n*=0, but was not apparent for *n* = 1/2 and 2/3. For *n* = 0 under *K =* 1/2, the *τ_c_* occurred at 10, 1, and 0.1 for *G* = 10^−1^, 10^0^, and 10^1^, respectively (Figure 4a–c). For *n* = 0 under *K =* 1/101, the *τ_c_* occurred at 1000, 100, and 10 for *G* = 10^−1^, 10^0^, and 10^1^, respectively (Figure 4d–f).

The profile of planar drugs in Figure 4 shows that release profiles in *τ* < *τ_c_* exhibited an initial burst in a short lag-time and a constant release rate thereafter. A zero-order release profile was identified for planar drugs before starting the surface depletion layer. It is sensible to define the *τ_c_* as the end point of the constant release rate region. Furthermore, since the release rate is nearly constant within the constant rate region, the starting point of the constant rate region, *τ_s_*, is, therefore, defined as the time in a release process first reaching the release rate at *τ_c_* with a deviation of less than 1%. The coverage of this zero-order release profile increased for a small *K* and *G*. A nearly zero-order release profile was also observed for cylindrical and spherical dispersed drugs when *K* and *G* were small. The linear relationship between the release fraction and time resulted from an interesting phenomenon in the profiles of drug concentration before the formation of the depletion layer. Due to the sustained exhausting dissolved drugs, un-dissolved drugs continued dissolving to supply the loss of dissolved drugs and brought about a constantly decreasing concentration of un-dissolved drugs with time [31]. However, the concentration profile of dissolved drugs remained nearly unchanged after the lag time required for the system to reach a quasi-steady state. This feature could cause the embedded drugs to be released at a constant rate [18].

Table 1 summarizes the characteristics of the constant release rate for different *K* and *G* values in Figure 4. Results showed that the constant release rate was evident for planar drugs with a high drug loading. The constant drug release rate increased with *G*, but the duration of constant release rate decreased with *G*. Although the constant drug release rate decreased with the *n* value, the constant drug release rate did not vary much for three *n* cases. The coverage of constant release rate in the whole release profile decreased obviously from *n* = 0 to 1/2 and 2/3. For *K* = 101, the coverage of constant release rate in the whole release profile for planar drugs reached 98.28%, 92.60%, and 66.88% for *G* = 10^−1^, 10^0^, and 10^1^, respectively. Nevertheless, the coverage dropped for cylindrical and spherical drugs.

### 3.4. Evaluation of Possible Deviation by Higuchi Approximation

**Figure 5:**
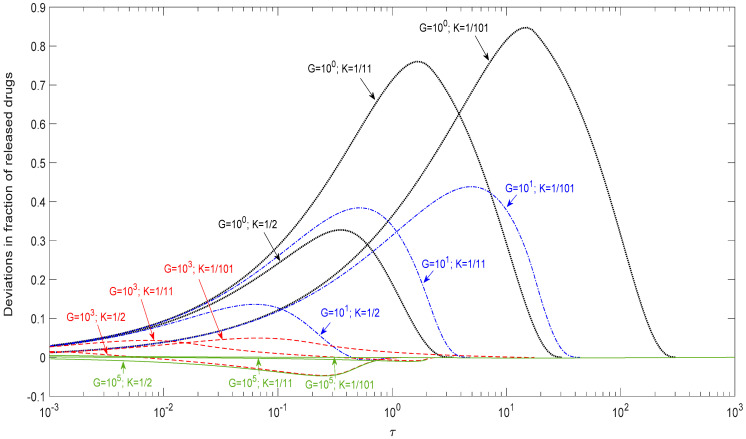
Deviations between Higuchi’s model and this present model for *n* = 2/3.

Higuchi’s model is a milestone to estimate the release of a drug-loaded particle, and nowadays is used as a correlation function. However, there is no available data for people to estimate the possible error that might be introduced due to the effect of the finite dissolution rate of the dispersed solid drug particle. In order to provide a reference tool for people to check up the possible error, Figure 5 examines the deviations between Higuchi’s model by Equations (13) and (14) and this present model for spherical drugs in different *K* (1/2, 1/11, and 1/101) and *G* (10^0^, 10^1^, 10^3^, and 10^5^) values. The deviation of released drugs was defined by subtracting the release fraction in this present model from Higuchi’s model. Positive deviation meant that the release fraction in Higuchi’s model was greater than in the present model. Therefore, the positive deviation revealed the overestimation of the release rate by Higuchi’s model. 

**Figure 6:**
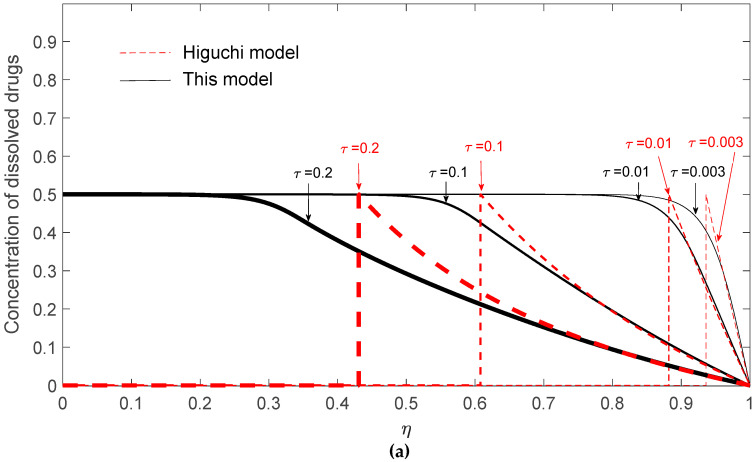

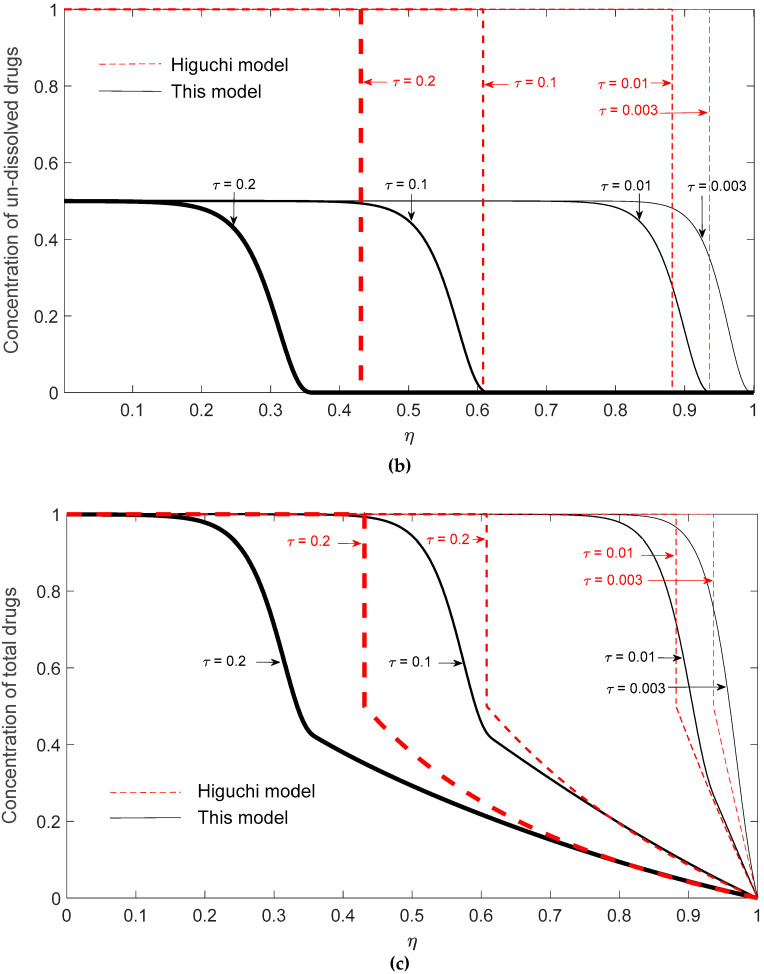
Radial drug concentration profiles in the matrix based on Higuchi’s model and this present model for *K* = 1/2 at a fixed *G* = 10^3^ for (**a**) dissolved drugs; (**b**) un-dissolved drugs; (**c**) total drugs.

To explain the possible negative deviation, the dynamic radial concentration profiles of dissolved drugs, un-dissolved drugs, and total drugs are demonstrated in Figure 6a–c, respectively, for *G* = 10^3^ and *K* = 1/2 at *τ* = 0.003, 0.01, 0.1, and 0.2. The fraction of remaining drugs can be obtained by integrating the sum of the radial concentration profiles of dissolved and un-dissolved drugs. In Figure 6c, it is clearly shown that the total drug concentration evaluated by Higuchi’s model is higher than that evaluated by the present model for *τ* = 0.1 and 0.2, suggesting that the remaining drugs in the matrix predicted by Higuchi’s model are higher than the present model and, therefore, a negative deviation occurs at these two time points.

These results can be explained from two aspects: one is the fast development of the surface depletion zone and the possible lower transient surface concentration gradient of Higuchi’s model. Firstly, although the initial release rate predicted by Higuchi’s model is always faster, the release rate might drop quickly due to the quick development of the surface depletion layer. Since the diffusional resistance is proportional to the square of travel distance, the increase of the surface depletion layer thickness should greatly reduce the drug diffusion rate. As shown in Figure 6b, the concentration profiles of un-dissolved drugs predicted by Higuchi’s model give a depletion layer thickness of ~0.07 and 0.12 at *τ* = 0.003 and 0.01, respectively, while a depletion layer thickness of ~0 and 0.07 is observed by the present model. This result suggests that the quickly built surface depletion layer might temporarily cause a negative deviation of Higuchi’s model. Besides, the negative deviation can also be explained by the concentration profiles of dissolved drugs as shown in Figure 6a. By closely examining the concentration profiles at the surface of the drug carrier, it is observed that the surface concentration gradient of dissolved drugs by the present model is higher than that by Higuchi’s model for *τ* = 0.003 and 0.01. Since the drug release rate is proportional to the surface concentration gradient of dissolved drugs, this result again suggests that Higuchi’s model might give a negative deviation during a dynamic release process.

Conceptually, the negative deviation of Higuchi’s model observed for the rare cases at low drug loading can be explained by the feature of its surface erosion type of drug release. In a surface erosion process, the remaining drugs are mainly allocated in the core region of the carrier. Meanwhile, in the present model, the undissolved drugs are more evenly consumed along radial direction. As a result, the un-dissolved drugs in a carrier with a finite dissolution rate can remain closer to the surface of the carrier matrix and continue to serve as a reservoir to maintain a higher surface concentration gradient of the carrier. For the case of low drug loading and with a saturated initial dissolved drug concentration, the effects of the distribution of remaining drugs in the carrier might be greater than the effect of a finite dissolution rate and, therefore, cause a transient negative deviation.

In Figure 5, this deviation was found to be prominent in the case of a small *G* value. In the early stage, the deviation was insensitive to the *K* value, but became prominent at the late stage. In general, the absolute deviation was small for a large *G* and large *K* value, i.e., a high dissolution rate and low drug loading such as *G* = 10^3^ and *K =* 1/2 in this study, which approached the basic assumption in Higuchi’s model. Table 2 summarizes the maximum deviations for the release conditions in Figure 5. As shown in the table, Higuchi’s model can approximately describe the release curves of *G* = 10^3^–10^5^ under *K* = 1/101–1/2 within a 5% error. Since the absolute deviations of Higuchi’s approximations were all smaller than 5% for *G* **≧** 10^3^, regardless of *K*, Higuchi’s model, therefore, afforded an acceptable description of the drug release profile when *G* **≧** 10^3^.

The practical usage of this proposed model will be limited if one has to perform the calculations by oneself for cases with specific *K* and *G* values. As a remedy to this disadvantage, we thus analyzed the deviations between Higuchi’s model and this present model and came up with results given in Figure 5 and Table 2. These results cover a wide range of *K* and *G* values and provide direct information for the possible maximum error one might have if the release curve is evaluated by the simple Higuchi’s model. For a case with a given tolerance on the deviation of drug release, one is thus able to justify the application of the simple Higuchi’s model for certain ranges of *K* and *G* values.

## 4. Conclusions

This study derived a more accurate model for drug release that considers a finite dissolution rate and different drug particle shapes from a spherical matrix. The effects of the dispersed drug shape on release profiles were investigated for the first time. Numerical solutions of planar drugs were compared with a short-time analytical solution and with the corresponding diffusion-controlled limits in Higuchi’s model to validate this release model developed herein. The results indicated that Higuchi’s model is suitable for a large ratio of dissolution/diffusion rate (*G*) and holds for a ratio of drug solubility/initial loading (*K*) as large as 1/2. A constant rate of release was found before the formation of the depletion layer for planar drugs, and a large portion of the release process could be described by the analytical solutions in the case of a small *G* value. As for the effects of drug shape, the rate of release decreased with the shape factor (*n*) and the variation diminished gradually with the increase in *G*. The deviation analysis revealed that Higuchi’s model could well predict the actual release within a 5% error for the case of *G* = 10^3^–10^5^ under *K* = 1/101–1/2.

## Figures and Tables

**Table 1:** The characteristics of the constant release rate for different *K* and *G* values in Figure 4.

*K*	*G*	Time Region of Constant Release Rate (*τ_s_*–*τ_c_*)	*n*	Release Rate		Coverage of Constant Release Rate (%)
				Range	Middle	
1/2	10^−1^	0.91–10	0	5.02 × 10^−2^–4.97 × 10^−2^	4.97 × 10^-2^	45.77
			1/2	4.84 × 10^−2^–2.65 × 10^−2^	3.75 × 10^-2^	33.17
			2/3	4.78 × 10^−2^–2.36 × 10^−2^	3.57 × 10^−2^	30.99
	10^0^	0.59–1	0	4.75 × 10^−1^–4.70 × 10^−1^	4.73 × 10^−1^	20.66
			1/2	3.77 × 10^−1^–2.84 × 10^−1^	3.31 × 10^−1^	13.36
			2/3	3.54 × 10^−1^–2.56 × 10^−1^	3.05 × 10^−1^	12.31
	10^1^	Negligible–0.1	0	-	-	-
			1/2	-	-	-
			2/3	-	-	-
1/101	10^−1^	0.91–1000	0	9.94 × 10^−4^–9.84 × 10^−4^	9.89 × 10^−4^	98.28
			1/2	9.94 × 10^−4^–5.19 × 10^−4^	7.57 × 10^−4^	73.92
			2/3	9.94 × 10^−4^–4.62 × 10^−4^	7.28 × 10^−4^	69.39
	10^0^	0.61–100	0	9.40 × 10^−3^–9.30 × 10^−3^	9.35 × 10^−3^	92.60
			1/2	9.38 × 10^−3^–5.21 × 10^−3^	7.30 × 10^−3^	71.29
			2/3	9.38 × 10^−3^–4.65 × 10^−3^	7.02 × 10^−3^	67.13
	10^1^	0.2–10	0	6.52 × 10^−2^–6.46 × 10^−2^	6.49 × 10^−2^	66.88
			1/2	6.49 × 10^−2^–4.57 × 10^−2^	5.53 × 10^−2^	53.98
			2/3	6.48 × 10^−2^–4.24 × 10^−2^	5.36 × 10^−2^	51.85

**Table 2:** The maximum deviations between Higuchi’s model and this present model for *n* = 2/3 (% fraction of released drug).

	*G*	10^0^	10^1^	10^3^	10^5^
*K*					
1/2		32.7	13.6	−4.6	−4.7
1/11		76.0	38.4	4.3	−1.1
1/101		84.7	43.9	4.9	0.5

## Nomenclature

*A*	drug surface area per volume unit sphere, 1/cm
*A_o_*	initial drug surface area per volume unit sphere, 1/cm
*C*(*r,t*)	dissolved drug concentration, g/mL
*C_a_*(*r,t*)	un-dissolved drug concentration, g/mL
*C_ao_*	initial un-dissolved drug concentration, g/mL
*C_s_*	solubility of drug in matrix, g/mL
*C_t_*	initial total drug concentration *C_t_ = C_ao_ + C_s_*, g/mL
*D*	diffusivity of drug in matrix, cm^2^/s
*G*	ratio of diffusion rate to dissolution rate *kA_o_r_o_^2^/D*
*K*	ratio of drug solubility to initial total drug concentration *C_s_*/*C_t_*
*k*	dissolution rate constant, 1/(cm^2^·s)
*n*	shape factor of a drug particle
*r*	radial coordinate from the left of a sphere, cm
*r_o_*	radius of a sphere, cm
*r^*^*	moving front, cm
*t*	time, s
*u*	unit step function
**Greek symbols**	
*φ*(*η*,*τ*)	dimensionless dissolved concentration *C*/*C_t_*
*ϕ*(*η*,*τ*)	dimensionless un-dissolved concentration *C_a_*/*C_t_*
*η*	dimensionless radius *r/r_o_*
*η^*^*	dimensionless moving front *η/r_o_*
*τ*	dimensionless time *Dt*/$r_{o}^{2}$
*τ_c_*	dimensionless critical time
*τ_s_*	dimensionless starting time of constant release rate region
